# Crowdsourcing, citizen sensing and sensor web technologies for public and environmental health surveillance and crisis management: trends, OGC standards and application examples

**DOI:** 10.1186/1476-072X-10-67

**Published:** 2011-12-21

**Authors:** Maged N Kamel Boulos, Bernd Resch, David N Crowley, John G Breslin, Gunho Sohn, Russ Burtner, William A Pike, Eduardo Jezierski, Kuo-Yu Slayer Chuang

**Affiliations:** 1 Faculty of Health, University of Plymouth, Drake Circus, Plymouth, Devon PL4 8AA, UK; 2 International Society for Photogrammetry and Remote Sensing, Commission IV - Geodatabases and Digital Mapping, WG IV/4 - Virtual Globes and Context-Aware Visualisation/Analysis, ISPRS Headquarters (2008-2012), National Geomatics Centre of China, Beijing 100048, PR China; 3 SENSEable City Lab, Massachusetts Institute of Technology (MIT), Cambridge, MA 02139, USA; 4 Institute for Geoinformatics and Remote Sensing, University of Osnabrueck, 49076 Osnabrueck, Germany; 5 School of Engineering and Informatics, National University of Ireland Galway, Ireland; 6 Digital Enterprise Research Institute (DERI), National University of Ireland Galway, Ireland; 7 Department of Earth and Space Science and Engineering, York University, Toronto, ON M3J 1P3, Canada; 8 International Society for Photogrammetry and Remote Sensing, Commission III - Photogrammetric Computer Vision and Image Analysis, WG III/4 - Complex scene analysis and 3D reconstruction, ISPRS Headquarters (2008-2012), National Geomatics Centre of China, Beijing 100048, PR China; 9 Pacific Northwest National Laboratory, Richland, WA 99352, USA; 10 InSTEDD (Innovative Support to Emergencies, Diseases and Disasters), Palo Alto, CA 94306, USA; 11 ITRI (Industrial Technology Research Institute), Hsinchu County 310, Taiwan

**Keywords:** Citizen Sensing, Sensors, Social Web Crowdsourcing, Twitter, Geo-mashups, Semantic Web, OGC Sensor Web Enablement, OGC Open GeoSMS, 3-D Visualisation, Natural User Interfaces, Public and Environmental Health, Crisis Informatics

## Abstract

'Wikification of GIS by the masses' is a phrase-term first coined by Kamel Boulos in 2005, two years earlier than Goodchild's term 'Volunteered Geographic Information'. Six years later (2005-2011), OpenStreetMap and Google Earth (GE) are now full-fledged, crowdsourced 'Wikipedias of the Earth' *par excellence*, with millions of users contributing their own layers to GE, attaching photos, videos, notes and even 3-D (three dimensional) models to locations in GE. From using Twitter in participatory sensing and bicycle-mounted sensors in pervasive environmental sensing, to creating a 100,000-sensor geo-mashup using Semantic Web technology, to the 3-D visualisation of indoor and outdoor surveillance data in real-time and the development of next-generation, collaborative natural user interfaces that will power the spatially-enabled public health and emergency situation rooms of the future, where sensor data and citizen reports can be triaged and acted upon in real-time by distributed teams of professionals, this paper offers a comprehensive state-of-the-art review of the overlapping domains of the Sensor Web, citizen sensing and 'human-in-the-loop sensing' in the era of the Mobile and Social Web, and the roles these domains can play in environmental and public health surveillance and crisis/disaster informatics. We provide an in-depth review of the key issues and trends in these areas, the challenges faced when reasoning and making decisions with real-time crowdsourced data (such as issues of information overload, "noise", misinformation, bias and trust), the core technologies and Open Geospatial Consortium (OGC) standards involved (Sensor Web Enablement and Open GeoSMS), as well as a few outstanding project implementation examples from around the world.

## State-of-the-art review

'Wikification of GIS (Geographic Information Systems) by the masses' is a phrase-term first coined by Kamel Boulos in 2005 [1], two years earlier than Goodchild's term 'Volunteered Geographic Information (VGI)' [2]. Six years later (2005-2011), Google Earth (GE) [3] is now a full-fledged, crowdsourced 'Wikipedia of the Earth' *par excellence*, with millions of users contributing their own content to it, attaching photos, videos, notes and even 3-D (three dimensional) models to locations in GE.

GPS (Global Positioning System) traces received from commuters via their Internet-enabled mobile devices can be used to generate real-time traffic updates, while geo-tagged, street-level audio samples recorded and uploaded by pedestrians using their location-aware smartphones can be aggregated to create citywide noise (pollution) maps for various times of the day and week [4]. Such applications are often referred to as 'crowdsourcing' or 'participatory sensing' applications, since they are capitalising on the power of the masses (or 'crowds') and relying on citizen participation to achieve their goals. They are becoming increasingly common these days, thanks to the rapidly growing affordability, availability and adoption rates in recent months and years of Internet-enabled and location-aware mobile devices, such as tablets and smartphones [5].

Geolocation-aware mobile crowdsourcing apps (short for applications, especially those designed to run on smartphones and tablets), such as Love Clean Streets [6], HealthMap's Outbreaks Near Me [7] and MedWatcher (drug safety surveillance) [8], and the San Ramon Valley (CA, USA) Fire Department app (a real-time, geo-aware lifesaving app that alerts 'citizen responders' trained in CPR (CardioPulmonary Resuscitation) as soon as a cardiac arrest has been reported to emergency services) [9], are leveraging the power of the Social Web ('Web 2.0') and smartphones to provide unprecedented levels of citizen engagement and participation in their local and wider communities.

Crowdsourced mapping examples (some are in real-time) include Sickweather [10], an online social health network for sickness (e.g., flu) forecasting and mapping; the crowdsourced real-time radiation maps [11] that made the news headlines following Japan's Fukushima Daiichi nuclear disaster in March 2011; and the 'Lunch Break' Web map [12], a poll and map launched by the Wimpfheimer-Guggenheim Fund for International Exchange in Nutrition, Dietetics and Management that provides a unique look at lunchtime eating patterns in North America. These apps and maps, freely available to the public online, are excellent examples of how crowd-enabled systems are revolutionising the way we tackle problems and allowing us to monitor and act upon almost anything, anywhere, in real-time.

GeoChat [13] and Ushahidi [14] are two open source platforms that enable the easy deployment of crowdsourced interactive mapping applications with Web forms/e-mail, SMS (Short Message Service) and Twitter [15] support. They can be freely downloaded and deployed on one's own server by anyone with the appropriate technical expertise or used as online services hosted by their respective platform providers (e.g., Crowdmap [16] is the hosted version of Ushahidi). Mobile apps are available for accessing Ushahidi on smartphones and tablets, e.g., for the Android platform [17].

In Cambodia, the Ministry of Health uses GeoChat for disease reporting and to send staff alerts and rapidly escalate response to potential outbreaks, while in Thailand, more than 900 facilities within the Hospital Network exchange information and get alerts to monitor influenza outbreaks in real-time from facilities across the country. GeoChat was also deployed during the 2010 earthquake relief efforts in Haiti to coordinate field teams' activities and provide remote support from outside the earthquake zone [13]. Ushahidi and Crowdmap can be similarly used to crowdsource and map crisis information from multiple data streams in real-time; see, for example, Ushahidi's crowdsourced map of the 2010 Haitian earthquake [18] and Thailand Flood Crisis Information Map at [19] (these latter two examples are also a good demonstration of Ushahidi's 'dynamic timeline' feature for tracking crowdsourced reports on the map over time and filtering the data by time). Maps of this kind provide situational awareness, which is essential in crisis management operations.

Real-time mining of indirectly (i.e., unsolicited, not obtained via a formal reporting form/not originally meant for posting to a specific crowdsourcing effort) self-reported and sousveillance information harvested from aggregates of Twitter and other social network feeds can offer useful data and insights about unfolding trends and emerging crowd behaviours at times of crises [20]. However, such (raw) data obtained from Social Web feeds often contain variable amounts of "noise", misinformation and bias (which can get further "amplified" through the viral nature of social media) and will usually require some advanced forms of filtering and verification by both machine-based algorithms and human experts before becoming reliable enough for use in decision-making tasks. WSARE (What's Strange About Recent Events)-type algorithms [21] and platforms such as SwiftRiver [22] (open source, provided by Ushahidi) can prove helpful in trying to filter the Social Web "firehose".

(Twitter can also act as a 'publish-subscribe' infrastructure for non-human sensors and smartphones to directly post (i.e., automatically push time, date and location-stamped sensor readings to Twitter) and consume (i.e., subscribe to, and data mine, sensor reading tweets) more crisp and objective observations about our physical world, using some agreed form of 'sensor tweeting standard' [23] or 'Twitter Annotations' (tweet metadata).)

**Figure 1:**
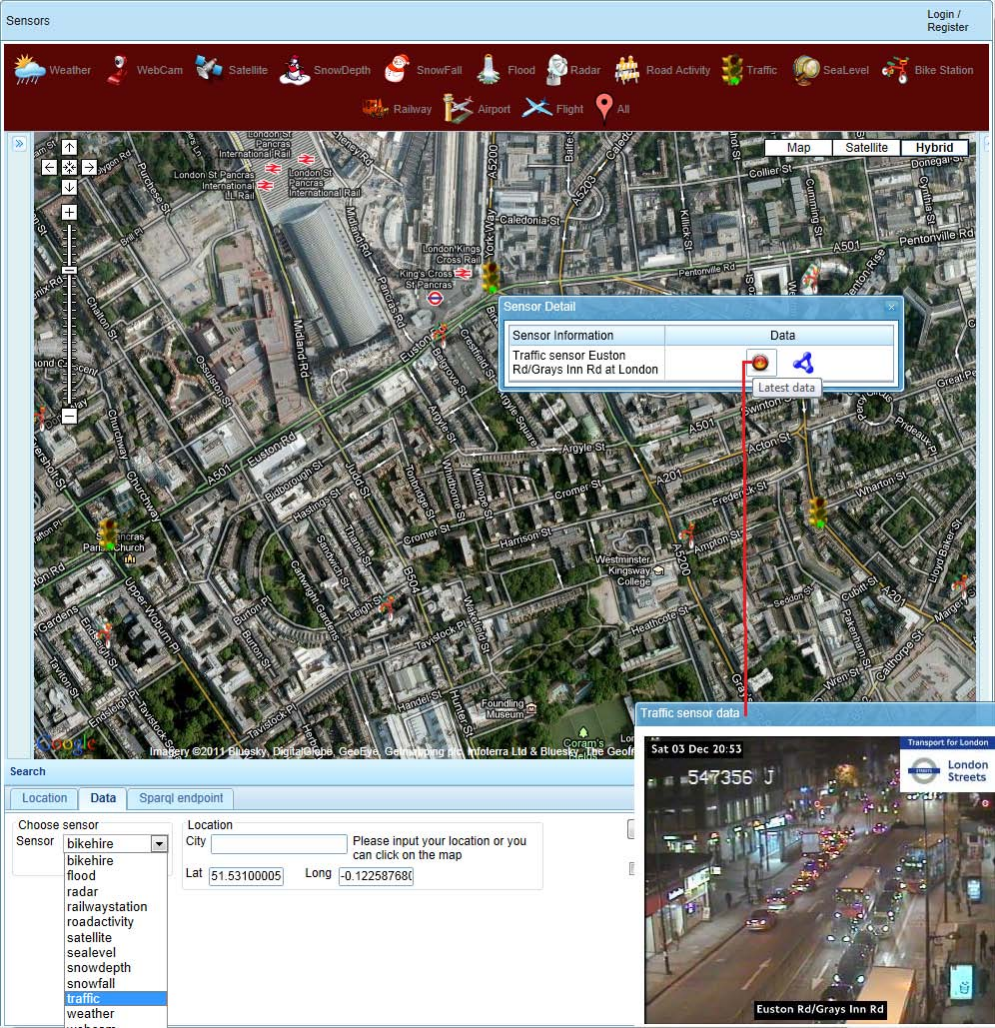
**Screenshot of DERI's LSM (Linked Sensor Middleware) live sensor geo-mashup**. Screenshot of the interface available at [21] showing near-real-time data obtained from a 'traffic sensor' in London, UK.

Besides human-triggered and/or generated input, more and more medical and other specialised sensor devices such as environmental and weather sensors, either fixed (e.g., at home, in buildings/rooftops or street furniture) or mobile (e.g., vehicle-mounted as in [24] or held/worn by commuting user), are being equipped these days with M2M (Machine-to-Machine) SIM (Subscriber Identity Module) cards or with Bluetooth or some other form of wireless communication with a suitable in-range relaying device, e.g., a user-held smartphone, to enable these sensors to send additional forms of non-human input to a remote aggregator service. These specialised sensor devices, usually operating within a network (or more than one network) of other distributed sensors, can automatically (or autonomously, depending on setup) gather and relay location (and/or person)-specific observations at intervals (scheduled or when required) or continuously, in real-time, to remote stations or 'situation rooms'. There (in the latter remote centres) data from multiple device sensors and/or human observers, covering one or more than one place, are collated, analysed, contextualised/triangulated (including with other non-sensor data) and summarised/visualised in different ways as appropriate, in real-time, near-real-time and/or for past points in time, e.g., DERI's live sensor geo-mashup [25,26] (Figure 1), to support monitoring, surveillance and decision-making tasks of various kinds.

While the 2-D (two-dimensional) user interface of DERI's live sensor geo-mashup (based on Google Maps) is very functional for 'situation room' purposes, improvements can still be made to realise the full vision of emergency/public health virtual situation rooms described in [27]. For example, the '3D Town' project at York University, Canada, is working to bring real-time sensing, tracking and surveillance of moving vehicles and people in outdoor and indoor city environments to static 3-D city models and 3-D virtual globes, thus making the latter truly dynamic by visualising live data on them [28], while colleagues at Pacific Northwest National Laboratory, USA, are developing future spatially-enabled work environments for emergency management, which they call 'Precision Information Environments' (or PIEs) [29]. PIEs aim at providing tailored access (i) to information from multiple data streams/sensors, and (ii) to analysis, simulation, decision support, and collaboration/communication capabilities. PIEs achieve this through novel interactions that adapt to the varying users (e.g., first responders, policy makers and the public) and phases of emergency management (from planning to response, recovery and mitigation) in distributed situation room and field settings (see concept video at [30]).

The Open Geospatial Consortium's (OGC) Sensor Web Enablement (SWE) standards allow developers to make all types of sensors, transducers and sensor data repositories discoverable, accessible and useable (for tasking, eventing and alerting) via the Web by defining service interfaces that enable an interoperable usage of sensor resources. A Sensor Web based on SWE services and standardised interfaces hides the heterogeneity of its underlying sensor network, as well as the complexity of its communication details and various hardware components, from the applications that are built on top of it, thus making the development and deployment of such applications a much easier task [31,32].

Another OGC standard, Open GeoSMS, enables the interoperable communication of location coordinates and content between different location-aware devices and applications using the extended Short Message Service (SMS), while maintaining human readability of the content [33]. Open GeoSMS has been implemented in Ushahidi and in the open source Sahana Disaster Management System [34] and has proven very useful in emergency and crisis management situations.

Following on from this condensed, bird's eye review of the field, the remaining sections of the paper will now shed some additional light on a select number of key issues, core standards and technologies, as well as a few outstanding project implementation examples related to the subject.

### A closer look at citizen sensing (participatory sensing) and 'human-in-the-loop sensing' in the era of the Mobile and Social Web

In 1999, before the advent of Foursquare [35], mobile Twitter clients or GPS and sensor-enabled phones, a somewhat prescient Neil Gross in Bloomberg Business Week said:

"*In the next century, planet earth will don an electronic skin. It will use the Internet as a scaffold to support and transmit its sensations. These will probe and monitor cities and endangered species, the atmosphere, our ships, highways and fleets of trucks, our conversations, our bodies-even our dreams *[36]."

This view of sensors as ubiquitous and being embedded into our everyday environment can be seen as an accurate description of human-sensor interaction today with the advancements in wireless sensor networks and with the huge growth in the use of mobile devices which contain multiple sensors. The unprecedented 96% growth in smartphone sales (in Q3 2010) [5,37] displays the availability and prevalence of these relatively cheap mobile sensing devices that enable Internet users to become sensing devices.

In the past ten years, we have seen the growth of online social networks and an explosion in user-generated content on the Web published from mobile devices to social platforms such as Twitter. Twitter is a microblogging platform founded in 2006, which by September 2011 had 100 million active monthly users and 400 million monthly visitors [38]. As of June 2011, Twitter processed 200 million posts (or 'tweets') per day [39]. In parallel with this growth of social networks, there has been a surge in sensor networks, many of which are also connected to the Internet, e.g., [40]. These usually consist of multiple static or inert sensors that capture certain readings from their environment whenever they are programmed to do so. In addition, many people are now carrying some form of sensor-laden device - a mobile phone, a tablet, a fitness device - from which sensor readings can also be retrieved. This is sometimes called 'human-in-the-loop sensing', but cars, animals and other moving entities can also incorporate sensors.

Cuff *et al. *propose that pervasive computing has moved from the laboratory into the natural environment [41], laying the foundations for Mark Weiser's concept of ubiquitous computing, which he sees as the "*availability of computers throughout the physical environment, virtually, if not effectively, invisible to the user*" [42,43]. Cuff *et al. *describe mobile devices as "*passive sensors that can silently collect, exchange, and process information all day long*" [41]. This style of sensing is called 'urban sensing' due to its suitability for large urban areas with high population density, but could be implemented in any environment. Campbell *et al. *view "*urban sensing as a departure from existing thinking on sensor networks because people are no longer just consumers of sensed data about some natural phenomenon or ecological process. Rather data about people are now sensed and collected such that the sets of producers and consumers of sensed data now overlap; people are 'in the loop' and may participate in both roles *[44]."

**Figure 2:**
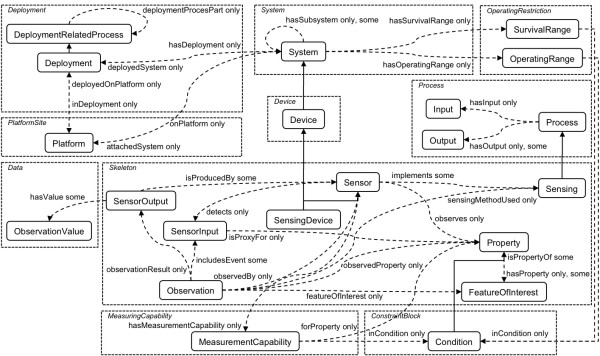
**SSN (Semantic Sensor Network) ontology**.

Cuff and colleagues raise two issues with urban sensing (which affect all human-in-the-loop sensing generally): bad data processing and the 'observer effect' [41]. Eradicating these issues or lessening their effect by verification of data with other sensor nodes/human input data can be accomplished but depends on the density of the network and existence of other related data. Chatzigiannakis *et al. *discuss technical challenges to pervasive applications in urban sensing, which include multiple data streams of varying data types, different skill/knowledge levels of users, and privacy and security of user data [45]. Some of these challenges can be overcome by using standardised sensor descriptions and models; the Semantic Sensor Network ontology (SSN) is an example of this and creates a domain-independent and full model for sensing applications by merging sensor-focused (e.g., SensorML [46], part of OGC SWE), observation focused (e.g., Observations and Measurements [47], part of OGC SWE), and system-focused models [48]. Figure 2 presents an overview of the classes and properties in the SSN ontology. The ontology representation of a sensor links together what it measures (the domain), the physical sensor (the grounding), and its functions and processing (the models). The SSN ontology does not try and describe every sensor and scenario but instead develops a general representation of sensors and a domain description; it relies on upper-level ontologies to define the domain and on an operation model that describes how the measurement is implemented [49].

Burke *et al. *assert that "*participatory sensing will task deployed mobile devices to form interactive, participatory sensor networks that enable public and professional users to gather, analyse and share local knowledge*" [50]. They envisage mobile devices equipped with cameras, GPS, and microphones acting as sensor nodes or as location-aware collection instruments. This idea of a mobile device wireless sensor network removes many of the barriers to implementing urban sensor networks. Barriers and challenges exist such as (i) initial cost of the nodes, (ii) the need to position/distribute the sensors appropriately, and (iii) implementing a network dense enough to build in redundancy if individual sensor nodes fail. Welsh examines issues with implementing wireless sensor networks such as random positioning (redundant in scientific experiments, as location is often a requirement and due to cost of nodes), the cost and size of nodes, and creating dense networks [51]. Density raises issues in other ways through redundancy and network capabilities - redundancy is beneficial, but network capabilities may be limited, so redundancy might not be possible to implement. Mobile cellular networks also suffer from this density challenge when a large amount of users using bandwidth can cause a flooding of the network. This network flooding can often occur during times of crisis as seen on 13 July 2011 in Mumbai, India, where after three explosions mobile networks were unusable by government officials and the general populace [52,53].

Burke *et al. *discuss the core network services in a participatory application, which include the concept of network-attested location and time, physical context using sensors (accelerometer, compass to work out direction device is facing), distance between devices/nodes, and context resolution control which controls the level of information shared by any one user or sensor. They foresee different applied areas for the research field in public health, urban planning, cultural identity (images with location, time, and context), and natural resource management, and state the goal of these services as placing "*users in the loop of the sensing process and aiming to maximise the credibility of data they collect*". They see these applications as an aid to researchers, policymakers and the public that "*use data to understand and persuade; higher quality data tend to generate more significant action and better understanding which leads to better planning, and policy decisions*" [50]. Citizen sensing and "slogging" (sensor logging) [54] concepts then emerge from this participatory sensing research where citizen sensors embedded in their environments report/analyse their surroundings.

Sheth describes users of the Internet or Web-enabled social community as citizens, and the ability to interact with Social Web ('Web 2.0') services can augment these citizens into citizen sensors, "*that is, humans as citizens on the ubiquitous Web, acting as sensors and sharing their observations and views using mobile devices and Web 2.0 services*" [55]. Goodchild discusses citizen sensing in the field of Volunteered Geographic Information (VGI) and sees citizens as a network of human sensors with over "*6 billion components, each an intelligent synthesiser and interpreter of local information. One can see VGI as an effective use of this network, enabled by Web 2.0 and the technology of broadband communication*" [2]. He sees each human sensor node as being "*equipped with some working subset of the five senses and with the intelligence to compile and interpret what they sense, and each free to roam around the surface of the planet*" [2]. Events, online social networks, or networks created more spontaneously by events can create citizen sensor networks - "*an interconnected network of people who actively observe, report, collect, analyse, and disseminate information via text, audio or video messages*" [55]. It is this 'human-in-the-loop sensing' combined with Social Web services and mobile computing that leads to the creation of citizen sensors and differentiates it from urban or participatory sensing. Sheth lists the advantages of 'human-in-the-loop sensing' as:

• Machines are good at symbolic processing but poor at perception;

• Machines are good at continuous, long-term sensing but humans can contextualise, discriminate and filter; and

• Learning, adapting, background knowledge, common sense and experience [55].

Humans continuously subconsciously and consciously sense, process, and induce inferences from events around them in real-time. Sense in this context is defined as one of the methods for a living being to gather data about the world: sight, smell hearing, touch, and taste [56]. Humans also leverage past experiences, background knowledge, and reasoning to extract meaning from often confusing or new experiences. Srivastava and colleagues define the following different participation roles of human-centric (or 'human-in-the-loop') sensing:

• (Humans as) information source;

• Measurement collection;

• Sensor data processing;

• Information sharing; and

• Information fusion and analysis [57].

These roles show that the human sensors perform processing and analysis of the collected data [57]. This pre-processing/processing of sensory data from experience/background knowledge is what differentiates our sensing capabilities from hardware sensors. Sensing can be defined as an operation of a sensor, the detection of a physical presence and the conversion of that data into a signal that can be read by an observer or an instrument [58]. In citizen sensing, a sensor is not necessarily a hardware sensor but can be a virtual sensor or a human interpreting sensory data. Sheth also envisages that microblogging platforms (where users can post short textual messages, Internet links, attached videos, and pictures) as low-effort publishing services are of particular interest to citizen sensing due to the large scale of users on platforms such as Twitter, which enables the user to post from mobile devices with minimum effort through mobile applications [55]. As microblogging lends itself to instantaneous updates, creation of data related to events around the world is posted before it can be reported on by more traditional media methods or even by blog or blog-like services. He uses the example of Twitter posts during the Mumbai terrorist attacks in November 2008, when Twitter updates and Flickr [59] feeds by citizens using mobile devices reported observations of these events in almost real-time [60].

These events are spatially, temporally, and thematically (STT)-linked; Sheth describes related events as situations. These events and series of related events can rapidly create networks of users posting information, but currently it can be difficult to track and collate all this information and make sense of all related posts. These events can be used to describe a model where, according to Westermann, events are "*the basic unit for organising data and for user interaction*" [61]. Jain defines this event-driven model as a "*human-centred computing system that will give users a compelling experience by combining quality content, carefully planned data organisation and access mechanisms*" [62]. These events can lead to *ad-hoc *spontaneous networks that are not necessarily socially interlinked but are event-connected, where such events are described in spatial, thematic, and temporal terms. These events as viewed by multiple citizen sensors can have different perspectives taken from the citizen's own assessment of the event, which can also influence the reporting. Kwak *et al. *discuss the role of Twitter as a social network or as a news media; their research shows that a retweet (where a retweet or 'RT' on Twitter describes an email-like forwarding mechanism practised by Twitter users to show the origin of the post/tweet) reaches an average of 1,000 users and this reach value is independent of the number of followers of the original poster [63]. Retweet trees show the spread of news and other interest pieces on Twitter and show the usefulness of Twitter as an event tracking mechanism and as a 'citizen journalism' platform. Sheth [64] describes how semantic annotation (using Resource Description Framework - in - attributes--RDFa [65]) of sensor data and then citizen sensor data [55] can aid metadata already embedded within user posts on Twitter and Flickr. This semantic annotation would assist thematic analysis and aid in disseminating information from informal SMS-style language. The building of semantic domain models about specific event types but in a generalised event model to describe, for example, natural disasters instead of specific types, and models to describe geographical locations would also aid in the language processing of such informal text. These general domain models would also remove the need to create more formal domain models that require agreement by specialised domain experts.

An example of a citizen sensing application, Twitris [66] is used to show STT analytics and how the system can be deployed to identify events [67]. Nagarajan *et al. *describe the challenges to developing citizen sensing platforms and gathering topically relevant data [68], as Twitter does not categorise users' posts, but instead Twitter's search API (Application Programming Interface) [69] is used with extraction of hashtags. The retrieved hashtags phrases are then used as seed keywords and seed keywords are generated by using Google Insights for Search [70]. This works on the presumption that high volume search terms describe events and are of high interest to users [68]. In this work hyperlinks to external content were ignored and only textual content was studied (hyperlink analysis was added in Twitris 2.0 [71]).

At present, GPS adds location to the data of the post made, but in the field of multi-sensor context awareness, researchers are always examining ways to augment devices with awareness of their situation and environment to add contextual understanding. As Gellersen *et al. *assert "*position is a static environment and does not capture dynamic aspects of a situation*" [72]. This idea can be applied to most single sensor data, but with multi-sensor context awareness, the diverse sensor readings are combined and then with processing, situational context can be derived. Mobile phones and tablet-style devices come equipped with an array of sensors and are positioned ideally to be the ubiquitous computing and communications devices in the near future. These sensors enable mobile phone sensing application domains such as healthcare and environmental monitoring [73] and can be utilised within the citizen sensing field.

### Bringing together the best of experts, crowds, and machines

**Figure 3:**
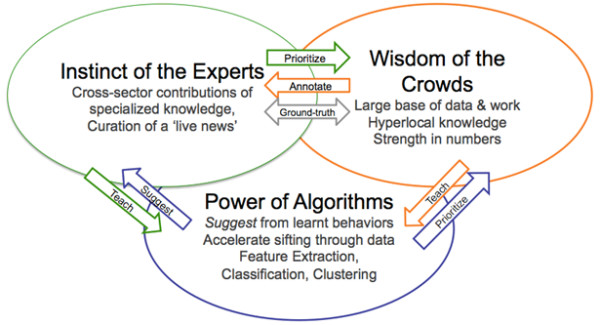
**Integrating experts, crowds, and signal processing algorithms**.

One needs to adopt concurrently a set of three practices for rapid analysis of large volumes of information: expert analysis, crowdsourcing (especially engaging networks of professionals in the health system, who have local knowledge and can do contributions from their perspectives, not only 'public crowds of citizens') and machine learning. Each of these three items provides useful data by itself, but used together, they are able to counter-balance each other's strengths and weaknesses, as shown in Figure 3.

Experts make decisions based on understanding data sources and the mechanics behind health events (e.g., transmission modes of diseases, time lags (incubation period) between infection and (onset of) symptoms, knowledge of the typical behaviour of patients, etc.). These experts can very rapidly generate a hypothesis about an unfolding event or validate, confirm or deny a certain characterisation. They also have in-depth knowledge about response protocols and sources of specialised data.

Experts, however:

• Are few in number and their time is precious;

• Are increasingly specialised, which allows them to make blink decisions correctly within their domain, but requires collaboration across other areas of expertise to contribute a 'complete picture' of a situation; and

• May have little local knowledge about local behaviours in an area where there may be an unfolding outbreak.

During the 2009 H1N1 influenza pandemic crisis, it was clear that the Internet and open data played a big role in capturing a ground truth of how people were reacting to the pandemic. Tamiflu (Oseltamivir, an anti-viral drug) availability, hospital queue lines, and impact on medical care provider stress levels were all extensively blogged, tweeted, and shared via listservs and other modes of communication. This provided greater quantity and quality of information than was expected.

Information from 'crowds' and communities of practice can be very useful, with the following considerations in mind:

• Twitter and other social media can disseminate both valid and invalid information [74]. Crowdsourced 'data' have a tendency to be resistant to nuance and correction; especially in social media, once a meme snowballs in the 'echo chamber', it can be very hard to correct (or 'counter-tweet') and the crowd is sometimes not so fast at changing course;

• Crowds often have no immediate way to discern truth from falsehood; what gets propagated is the 'popular' opinion shaped by the most prominent personalities, beliefs and agendas of the individuals in the 'crowd'. Technically-savvy citizens also introduce an interesting skew in the velocity with which their ideas spread and get shared; even more if there are political agendas involved. For example, tools to visualise H1N1 spread became popular more on the basis of visual appeal than quality of the data or the processes used to generate or validate them (but opinions are not all too bad, and opinion mining and sentiment analysis algorithms still have an important place in some Social Web mining and surveillance applications [75]);

• Crowds are prone to add opinion to data; which sometimes sticks more than the credible data themselves. Separating opinion and credible data through expert interpretation and curation, both centralised and decentralised, is important (in de-centralised curation, specific statements of fact expressed or extracted from citizen-generated information are validated, refuted, and expanded by the citizens themselves in a more distributed system (*cf*. concepts of peer reviewing and darwikinism [76]); and

• There are a few agencies (e.g., [77]) that have organisational or procedural channels specifying how to aggregate and incorporate information emerging on the Internet in decision making.

As for algorithms, in the business and finance sector, Derwent Capital and Bloomberg (with WiseWindow) are said to be already using Twitter mining algorithms in making useful stock market predictions with good accuracy [78,79]. Machine learning algorithms can be used to extract useful information from large amounts of data. Most machine learning applications in health use supervised learning, a subset of machine learning that uses 'feedback' (from humans or other systems) to improve its learning over time. Common uses of machine learning include:

• Feature extraction: For example, extracting ICD-9 (International Classification of Diseases) codes and references to syndromes or diseases from text, as well as interpreting text to infer time, location, people, etc. referred to in it;

• Classification: Being able to classify, group or tag information based on some explicit or unknown criteria;

• Clustering: Machines can process vast amounts of data and present correlations and proximities that escape the human eye and brain, sometimes discovering non-obvious correlations between variables; and

• With large amounts of data available, it is not even necessary to have a deep understanding of the relationships within the data themselves: machines can on their own distil the noise from the relevant correlations through successive optimisation.

However, machine learning has shortcomings of its own, when used in isolation:

• Some algorithms process signals in a way that is more specific than sensitive, meaning that important signals may be missed (false negatives). A combination of algorithms is important to draw different types of events and event features from undifferentiated data; understanding which algorithms, through experience, is essential;

• Algorithms need to be thoroughly validated and tested; and each new situation may place a new challenge on a given algorithm that may not allow the assessment of the quality of the algorithm in real-time;

• Algorithms need data to train and feedback to learn. Sometimes this learning phase makes it hard to get value 'out of the box';

• Humans have a tendency to become "lazy" over time and experience with accepted algorithms, where over-dependency and improper cross-checks of an algorithm's results may result in missed or misinterpreted signals;

• Low social acceptance in some cultures of systems that do not function in a way that is predictable or describable by a human; and

• Past misuse of machine learning with little understanding of the cognitive science underpinnings of human communication has led users to fear (and AI (Artificial Intelligence) experts to boast) that an algorithm could be put on par with a human. State-of-the-art cognitive theories (Winograd, Flores, etc.) show that commitments (statements of fact) can only be provided by humans; and therefore provide a better framework for understanding where machine learning fits. In simpler terms: - Should an algorithm declare a health emergency? No - because there is no commitment behind it. Can an algorithm help present data to an expert or authority with 'suggestions' and 'red flags', and then the authority can declare a health emergency? By all means, and this is a smarter design.

### Standardisation enables interoperability: OGC Sensor Web Enablement Initiative and Open GeoSMS

The vision of pervasive sensing through 'citizens as sensors' poses considerable challenges in terms of interoperability involving data formats, service interfaces, semantics and measurement uniformity. Thus, one key prerequisite to achieve this vision is the broad usage of open sensor standards.

**The Sensor Web Enablement Initiative (SWE) **by the OGC (Open Geospatial Consortium) has recently gained importance through its maturity and its broad support from research and industry. SWE seeks to provide open standards and protocols for enhanced operability within and between multiple platforms and vendors. In other words, SWE aims to make sensors discoverable, query-able, and controllable over the Internet [80]. Currently, the SWE family consists of seven standards:

#### • Sensor Model Language (SensorML)

This is a general schema for describing functional models of the sensor. It provides an Extensible Markup Language (XML) schema for defining the geometric, dynamic and observational properties of a sensor. Thus, SensorML serves for discovering different types of sensors, supporting processing and analysis of the retrieved data, as well as the geo-location of observed values. Information provided by SensorML includes observation and geometry characteristics, as well as a description and a documentation of the sensor, and a history of the component's creation, modification, inspection or deployment.

SensorML aims to provide descriptions of sensors and sensor systems for inventory management, to supply sensor and process information in support of resource and observation discovery. Furthermore, SensorML aims to allow for geo-locating observed values (measured data), to provide performance characteristics (e.g., accuracy, threshold, etc.) and to offer an explicit description of the process, by which an observation was obtained.

An important capability of SensorML that has to be mentioned considering the background of this paper is the formation of sensor classes, i.e., sensing devices with the same properties. In SensorML, a sensor array defines a set of devices of the same type at different locations, whereby a sensor group describes several sensors that operate together to provide one collective observation.

#### • Observations & Measurements (O&M)

This is the counterpart to SensorML in the field of the actual study of phenomena. That is, it provides a description of sensor observations and measurements in the form of general models and XML encodings. The term observation is defined as "*an action with a result which has a value describing some phenomenon*". The O&M standard labels several terms for the measurements themselves, as well as for the relationship between them, whereby the extent is limited to measurement results, which are expressed as quantities, categories, temporal or geometrical values, as well as arrays or composites of these. Naturally, the monitored values normally require a reference system to enable a meaningful interpretation and further processing by providing a context for the results [81].

It shall be stated that despite OGC's tendency towards using the geographical position as the central and connecting element in geospatial standards, the location parameter is considered a regular measurement within O&M. This means that the position is equipollent with other measurands such as time, air temperature or satellite images.

#### • Transducer Model Language (TML)

Generally speaking, TML can be understood as O&M's pendant or streaming data by providing a method and message format describing how to interpret raw transducer data.

#### • Sensor Observation Service (SOS)

**Figure 4:**
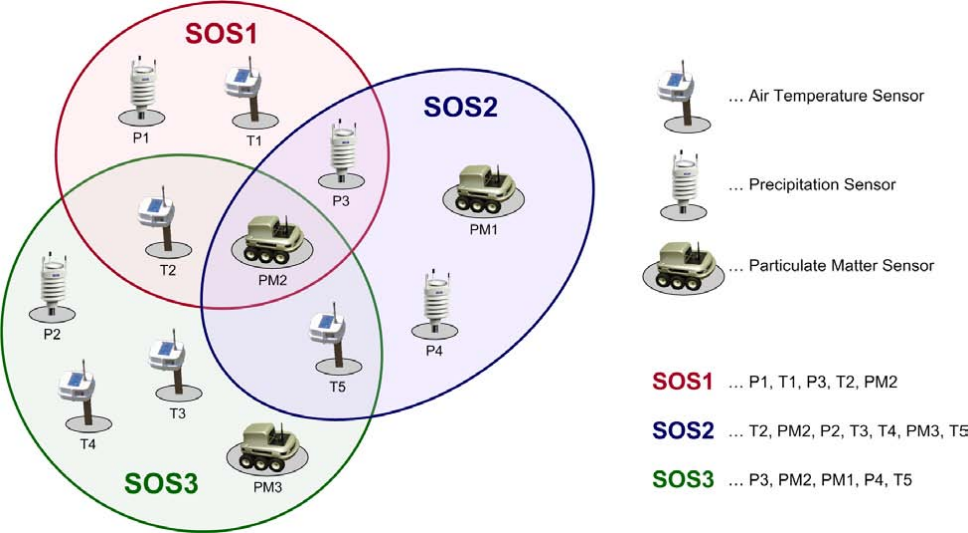
**SOS (Sensor Observation Service) general architecture (adapted from **[82]).

This component provides a service to retrieve measurement results from a sensor or a sensor network. In other words, the SOS groups a collection of possibly heterogeneous sensors, as illustrated in Figure 4[82], and provides their measurements via a standardised service interface.

The SOS specification defines the operations offered by a specific sensor, whereat the minimum collection of methods comprises GetCapabilites, DescribeSensor and GetObservation, which return information about the observations and measurements supported by the SOS. The kinds of data provided by a sensor and the sensor types themselves can be fetched from a sensor registry. SOS references the O&M specification for encoding sensor observations, and the TransducerML and SensorML specifications for modelling sensors and sensor systems.

#### • Sensor Planning Service (SPS)

This component provides a standardised interface for collection assets and aims at automating complex information flows in large networks. This requires the support of various capabilities, as well as different request processing systems, as described below.

The OGC recommendation defines interfaces for requesting information containing the capabilities of a SPS, for retrieving the practicability of a sensor planning request, for determining the status of such a request, as well as for submitting, updating or cancelling a sensor planning request [83].

Thus, SPS can be seen as the implementation of a bi-directional communication between a client and the sensor network. The client can send instructions to the network, which can be triggered by different kinds of events. One possibility is that a system administrator instructs the sensor network to send measurement values every five minutes instead of every hour in case a water level threshold is exceeded. Another commonly used sample functionality is the control of a remote camera to change orientation or focus.

#### • Sensor Alert Service (SAS)

The OGC SAS specifies interfaces (not a service in the traditional sense) enabling sensors to advertise and publish alerts, including according metadata. SAS can be used by clients to subscribe for sensor data alerts with some spatial and property-based constraints. Also, sensors can be advertised to the SAS allowing clients to subscribe for the sensor data via the SAS. SAS, which is currently in its version 0.9.0, is not released as an official OGC standard [84].

In the SAS context, 'alerts' are not only understood in the classic meaning of the word, i.e., an automatic signal or notification indicating that an event has fired (e.g., a message in case of threshold exceedance), but in a broader context. Alerts are defined as 'data' sent from the SAS to the client, which may as well comprise alerts/notifications (e.g., OGC Web Notification Service (WNS)) as observational data (measurements matching pre-defined criteria).

SAS uses the Extensible Messaging and Presence Protocol (XMPP) for the delivery of sensor notifications. Thus, SAS leverages an XMPP server, which can be embedded directly in the SAS or act as a separate service. SAS notifications are provided via a Multi User Chat (MUC) for each registered sensor. To receive notifications, a client has to join the specific MUC.

#### • Web Notification Service (WNS)

The last part of the SWE model is the Web Notification Service (WNS), which is a service enabling a client to perform asynchronous dialogues, that is message exchanges, with other services. This process is especially expedient when several services are required to comply with a client's request, or when an according response is only possible under considerable delays.

Principally, the service comprises two kinds of communications, the one-way-communication and the two-way-communication. The former, also known as simple WNS, for which users have to be registered, can be realised via the transmission of messages over e-mail, HTTP (Hypertext Transfer Protocol), Short Message Service (SMS), instant messaging, telephone, letter or fax. The latter system, viz extended WNS, is able to receive user notification responses [85].

Furthermore, sensor Web registries play an important role in sensor network infrastructures. However, they are not decidedly part of SWE, as the legacy OGC Catalogue Service for Web (CSW) is used.

The registry serves to maintain metadata about sensors and their observations. In short, it contains information including sensor location, which phenomena they measure, and whether they are static or mobile. Currently, the OGC is pursuing a harmonisation approach to integrate the existing CSW into SWE by building profiles in ebRIM/ebXML (e-business Registry Information Model).

Summarising, SWE aims at enabling the discovery and querying of sensor systems, observations, and observation procedures over the Internet. This process comprises determination of a sensor's capabilities and quality of measurements, access to sensor parameters that automatically allow software to process and geo-locate observations, retrieval of real-time or time-series observations and coverages in standard encodings, tasking of sensors to acquire observations of interest, and subscription to, and publishing of, alerts to be issued by sensors or sensor services based upon pre-defined criteria.

The OGC is developing SWE in tight coordination with other geospatial standards for data representation, provision and processing. This enables simple integration of sensor networks into existing Spatial Data Infrastructures (SDI) and data analysis systems by providing standardised service interfaces and APIs (Advanced Programming Interfaces). This development is of particular importance for achieving the far-reaching vision of 'citizens as sensors' in time-critical scenarios, such as emergency management and public health monitoring.

**Figure 5:**
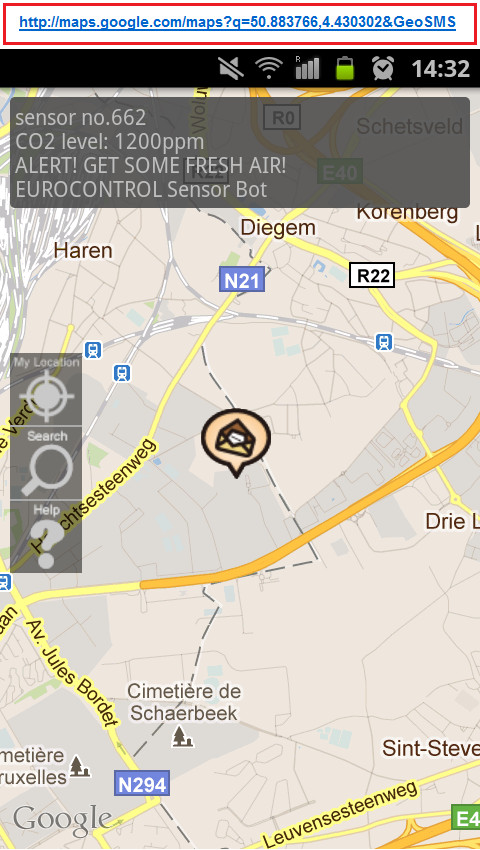
**Smartphone displaying details of an Open GeoSMS notification and incident location on Google Maps**.

**The OGC Open GeoSMS **is developed by ITRI (Industrial Technology Research Institute, Taiwan) for exchanging location information via the common mobile service, SMS [33,86]. Open GeoSMS can be composed with a mobile phone application, by retrieving GPS data and then embedding the geo-location coordinates in an SMS message. An example of an Open GeoSMS message is shown in Figure 5.

For an SMS notification to be compliant with the Open GeoSMS specification, the following criteria have to be fulfilled:

1. The first line of the SMS has to be a URL (Uniform Resource Locator)--see top of Figure 5;

2. This URL has × and y coordinates as the first two parameters;

3. This URL ends with '&GeoSMS'; and

4. Optional text can be appended for further description, e.g., of an (emergency) event at the specified geo-location and/or sensor readings at that location.

Mobile phones can be classified into three types for Open GeoSMS purposes:

• *A feature phone that allows no application to be installed*: Because this is an SMS message, almost every mobile phone can receive Open GeoSMS notifications similar to the one shown in Figure 5. The notifications are also readable by humans and users can easily understand that it is a CO2 level alert (to use the same example in Figure 5);

• *An online smartphone*: In the example presented in Figure 5, the user can simply click on the URL to access Google Maps service. The URL in Open GeoSMS can point to any given service provider (i.e., not necessarily Google Maps), so various kinds of information and services can be accessed via this URL. Of course, the application on the phone can also parse every incoming SMS, and once an Open GeoSMS is detected, coordinates are retrieved and further action, such as raising a warning or Standard Operation Procedure (SOP), is triggered; and

• *An offline smartphone*: Because the application on the phone can parse and get the coordinates in the Open GeoSMS message, offline maps such as OpenStreetMap [87] can be used to display the sent (incident) location, for those users who do not or cannot have data (Internet) access.

**Figure 6:**
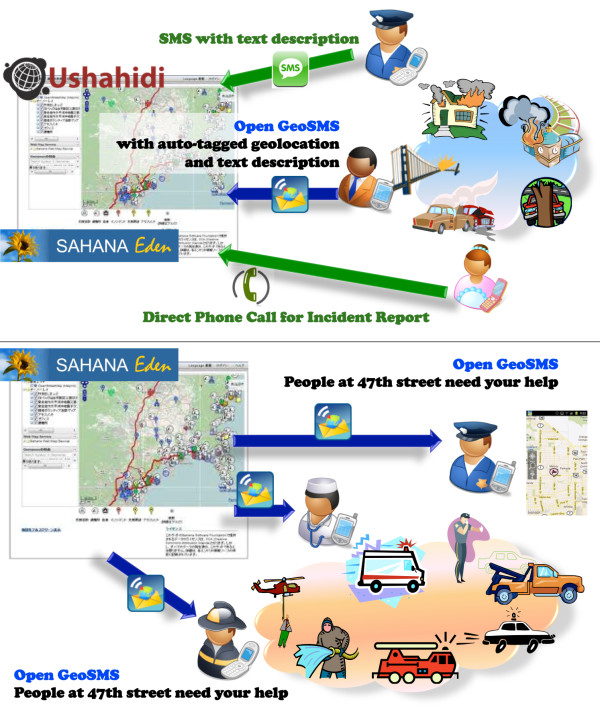
**Open GeoSMS is used in Ushahidi and in Sahana Disaster Management System**.

By overlaying the message text (incident details) on the map (Figure 5), Open GeoSMS presents clear information to users, including both geo-location and sensor information. Open GeoSMS can prove extremely helpful in public health emergency notification and management operations, since it works on almost any kind of mobile phone supporting SMS. It is an open standard that aims at enabling interoperability among different platforms. Figure 6 shows how Open GeoSMS has been implemented in Ushahidi [14] and in Sahana Disaster Management System [34] for incident reporting in emergency and disaster management operations. People reporting an incident can (still) make an ordinary phone call or send a conventional SMS message to the emergency services handling such situations, but with Open GeoSMS, geo-tagged SMS reports can significantly shorten the processing time for incident reports (and possibly save more lives by doing so). Open GeoSMS can also be used for task assigning and dispatching during disaster management operations (see bottom half of Figure 6).

### Common Scents - mobile pervasive sensing using bicycle-mounted sensors

The Common Scents project aims at providing fine-grained air quality data to allow citizens and urban decision-makers to assess environmental conditions instantaneously and intuitively. The goal is to provide real-time information on urban processes to support short-term decisions at multiple levels, from personal to governmental. To achieve this goal, the SENSEable City Laboratory at the Massachusetts Institute of Technology, USA, built up a mobile sensor network of bicycle-mounted environmental sensors to realise the vision of 'citizens as sensors'.

Within the Common Scents project the term 'real-time' is not defined by a pre-set numerical time information, but layers have to be created in a timely manner to serve application-specific purposes. For instance, an update on traffic conditions does not have to exceed a delay of a couple of minutes when this information is used for navigation instructions, whereas a 30-minute update interval can well be sufficient for short-term trip planning.

**Figure 7:**
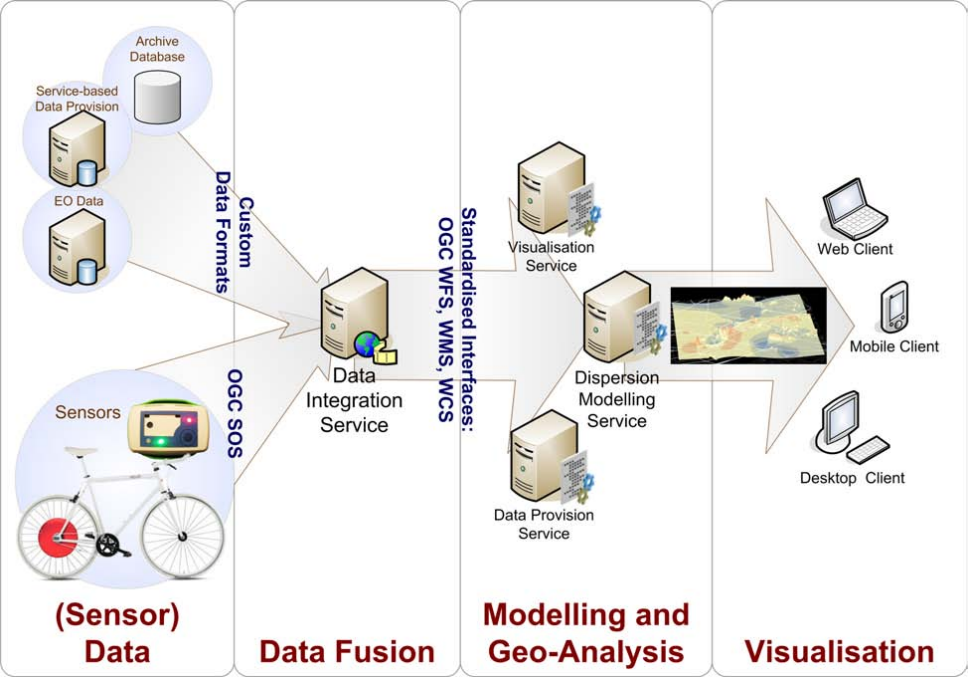
**Common Scents technical architecture**. WFS: Web Feature Service; WMS: Web Map Service; WCS: Web Coverage Service.

The Common Scents system architecture is based on the Live Geography infrastructure [88], which proposes a portable and open-standards-based monitoring infrastructure, including components for sensor data provision, sensor fusion, real-time data analysis, and user-tailored information visualisation. The approach accounts for different design principles such as Service Oriented Architectures - SOA, modular software infrastructures, and component-based development. This ensures flexibility, reusability and portability of the components and the overall infrastructure. Figure 7 shows the monitoring architecture and the standardised service interfaces that are used to connect the different components in the workflow of the Common Scents implementation.

According to principles of SOA and sustainable infrastructure development, a data collection, processing and information provision architecture was conceived and implemented, covering the whole process chain from sensor network development via measurement integration to data analysis and information visualisation, as shown in Figure 7. Hence, this infrastructure can potentially serve as the architectural bridge between domain-independent sensor network developments and use case specific requirements for end user tailored information output for public health monitoring.

The modules of the workflow shown in Figure 7 are separated by several interfaces, which are defined using open standards. These primarily include the Sensor Web Enablement (SWE) initiative that aims to make sensors discoverable, query-able, and controllable over the Internet, as described elsewhere in this article.

**Figure 8:**
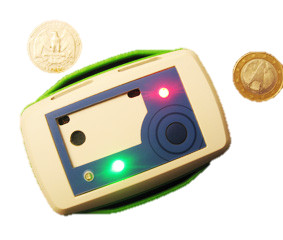
**Sensaris City Senspod**.

*Real-time air quality analysis using mobile bicycle-mounted sensors*: One pilot experiment in the Common Scents project was conducted in the city of Copenhagen, Denmark. Ten bicycle mounted sensors were deployed as a mobile sensor network to collect environmental data together with time and the geographic location, velocity and acceleration using GPS. In this experiment of ubiquitous mobile sensing, the Sensaris City Senspod (Figure 8) has been used, a relatively low-cost sensor pod. The deployment in Copenhagen was a combined effort of the MIT SENSEable City Laboratory and Københavns Kommune, Denmark.

The Sensaris City Senspod is a sensing device for environmental parameters that is dedicatedly designed for use in urban environments [89]. It collects CO, NOx, noise, air temperature and relative humidity, together with the geographic position and time via GPS. The City Senspod has proven to be a good choice for pervasive sensing because of its relatively low price and acceptable measurement accuracy. However, the City Senspod shows a few drawbacks including a minimum operational temperature of 5°C and its communication via Bluetooth, which is not the optimum transmission technology when aiming for maximum energy efficiency in the context of pervasive monitoring.

To comply with the standardised infrastructure of Common Scents system architecture, several standardised services were implemented on top of the sensor network, in accordance with the Live Geography approach. For data access, a Sensor Observation Service (SOS) was developed to supply measurement data in the standardised O&M format. It builds the O&M XML structure dynamically according to measured parameters and filter operations. To generate alerts, e.g., in case of exceedance of a threshold, an XMPP (Extensible Messaging and Presence Protocol)-based Sensor Alert Service (SAS) was implemented. It is able to detect patterns and anomalies in the measurement data and generate alerts and trigger appropriate operations such as sending out an email or a text message, or to start a pre-defined GIS analysis operation.

The pilot deployment itself has been carried out in the course of the Copenhagen Wheel project, which has been initiated by the MIT SENSEable City Lab [24,90]. This project was officially presented in Copenhagen on 15 December 2009 in the course of the 15^th ^Conference of the Parties during the 2009 United Nations Climate Change Conference meeting. The Copenhagen Wheel is a specially designed bicycle to capture energy dissipated while cycling and braking, to store it in an in-wheel battery and support the cyclist on demand through a small electrical engine.

In the Common Scents project, Sensaris City Senspods have been attached to the bicycles, capturing information about carbon monoxide (CO), NOx (NO + NO2), noise, ambient temperature, relative humidity, in addition to position, velocity and acceleration. The environmental sensors were originally intended to be placed within the hub of the bicycle wheel; however due to logistical pressure, they were placed on bicycles ridden by couriers in Copenhagen going about their normal daily routine. Ten cycles were instrumented and tested over 2 December 2009. It is believed that this was the first time multiple mobile sensors had been used in the field with such a large variety of environmental sensors on a city-wide scope.

The analysis component, which processes the collected data, performs a spatial Inverse Distance Weighting (IDW) interpolation on temperature measurements, which will be used in further research efforts for correlation operations with emission distribution or traffic emergence, and for the detection of urban heat islands. It has to be stated that IDW is presumably not an optimised algorithm for drawing conclusions from point measurements to a city-wide scale. In a future effort, an accurate urban dispersion model will be integrated into the analysis.

**Figure 9:**
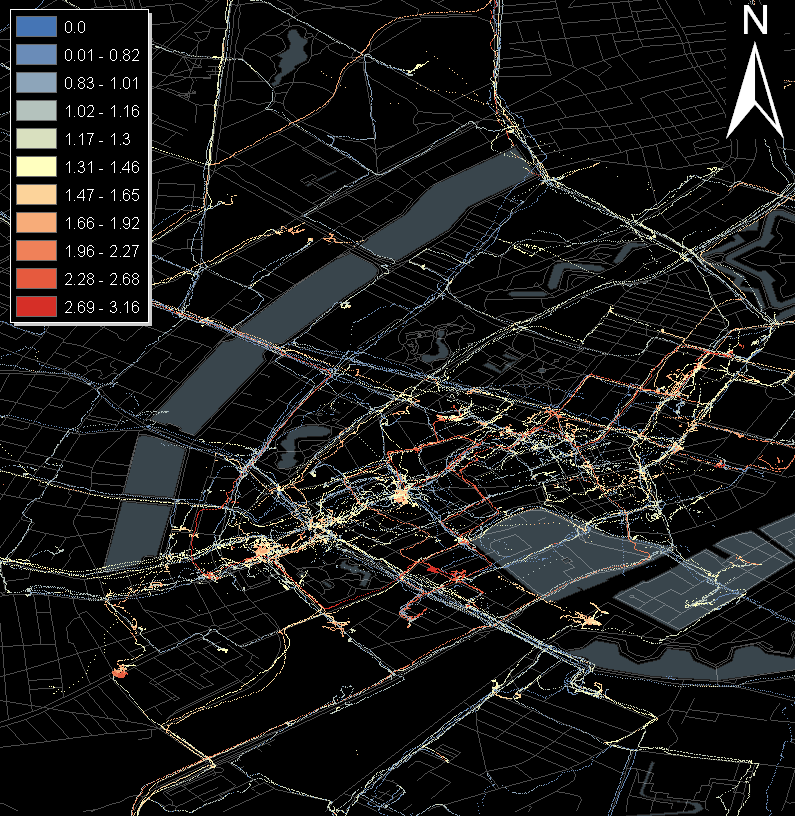
**Mobile CO measurements in the city of Copenhagen (December 2009)**.

In the field trial, the processing module analyses the CO, NOx, noise temperature and relative humidity distributions throughout the city of Copenhagen. The CO map containing the GPS traces, which figuratively re-draw the urban street network, is shown in Figure 9. A first qualitative analysis of the mobile measurements shows that there are strong correlations between ambient temperature, CO and NOx values. Further preliminary outcomes show that both CO and CO2 are undergoing very high temporal and spatial fluctuations, which are induced by a variety of factors including temperature variability, urban topography, time during the day, the 'urban canyon' effect, traffic emergence or 'plant respiration' - the fact that plants release major amounts of CO2 overnight.

Future research will include the investigation of direct correlations between pollutants, environmental measurements and traffic emergency. It is well known that CO is a measure of the efficiency of combustion in vehicles that may be used to reflect changing driving patterns and the sensitivity of air quality to larger scale environmental features such as wind speeds over the city. However, the detailed interplay of these parameters still has to be investigated in a next step. Especially CO values measured in the Copenhagen pilot have to be normalised over humidity and temperature to perform further quantitative (absolute amounts) and qualitative (impact on public health) analysis.

Concluding, it can be stated that the Copenhagen experiment was an important step toward the realisation of pervasive monitoring by the use of mobile sensors. Particularly, it has been shown that the vision of 'citizens as sensors' for environmental monitoring and public health is technically and methodologically feasible.

### LSM (Linked Sensor Middleware) - a geosemantic sensor mashup

Linked Stream Middleware (LSM) [26], a platform developed at the Digital Enterprise Research Institute (DERI) at National University of Ireland Galway, Ireland, that brings together the live real world sensed data and the Semantic Web [91] in a unified model is an example of a large scale sensor platform that if combined with Social Web data could provide a comprehensive insight into the domain of citizen sensor data. A sample LSM deployment is available at [25] and currently displays data from over 100,000 sensors around the world (Figure 1). The interface uses a map overlay to query and display sensor information. Several types of sensor data are available, such as flight status, weather, train/bus arrival times, street cameras, sea level monitors, etc. The history of the data produced by a particular source is available and downloadable in Resource Description Framework (RDF) format [92].

**Figure 10:**
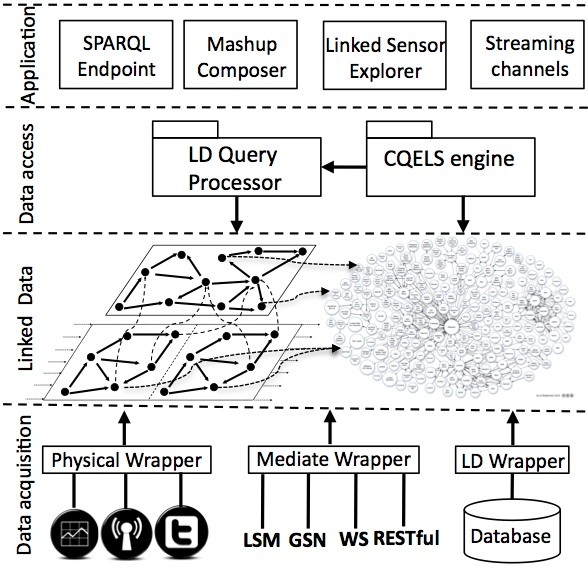
**Layered architecture of the LSM (Linked Sensor Middleware) platform**. 'RESTful' refers to conformance to REST (REpresentational State Transfer) constraints.

**Figure 11:**
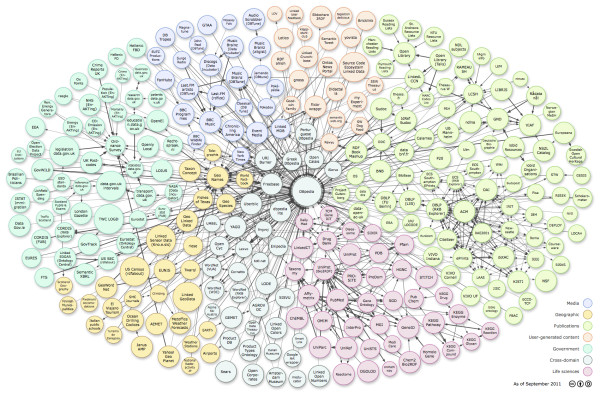
**'Linking Open Data' datasets as of September 2011**. Linking Open Data cloud diagram by Richard Cyganiak and Anja Jentzsch. A version of the same diagram with clickable hyperlinks is available at [95].

The LSM architecture, as shown in Figure 10, is divided into four layers. The Data Acquisition Layer provides three wrapper types: Physical Wrappers that are designed for collecting sensor data from physical devices; Linked Data (LD) Wrappers that expose relational database sensor data into RDF; and Mediate Wrappers, which allow for collection of data from other sensor middleware such as Global Sensor Networks (GSN) [93], Pachube [94], and the sensor gateway/Web services from National Oceanic and Atmospheric Administration (NOAA) [95]. The Linked Data Layer allows access to the Linked Sensor Data created by the wrappers but links to the Linking Data cloud (a subset of the Linking Open Data cloud [96] shown in Figure 11). The Data Access Layer provides two query processors, a Linked Data query processor and the Continuous Query Evaluation over Linked Streams (CQELS) engine [97], and exposes the data for end-users or machine-users. The fourth layer, the Application Layer, offers a SPARQL (SPARQL Protocol and RDF Query Language) [98] endpoint, a mashup [99] composer, linked sensor explorer, and streaming channels [92].

The LSM system is of interest in the area of citizen sensing because it implements an architecture that could be used for large-scale citizen sensing projects. As mentioned before, over 100,000 sources are available to the current running implementation of LSM. Scalability and reliability are important factors when designing any system; but when collecting real-time stream data (i.e., from Twitter and other social media feeds or from sensor feeds), uptime of a system becomes critical and so does its graceful scaling up to process the data in real-time. Transforming the sensor data into a standard format such as RDF also allows for standard sensor descriptions using the SSN ontology as described earlier. Some research in the area of citizen sensing has tried to leverage the power of the SSN ontology to describe sensors on mobile devices for rural transportation projects [100] and in emergency reporting applications on microblogging platforms [101].

Annotating microblog posts with sensor data could add context and remove some of the ambiguity of the short SMS-style languages commonly used on microblogging systems and could aid the processing of these texts for information extraction using natural language processing. (The concepts of annotations and machine-readable metadata that "travel" with a tweet (without using any of the 140 characters that are reserved for the tweet text message) were the basis of the not-yet-released (as of Q4 2011) 'Twitter Annotations' feature [102] and related work by Bottlenose [103], and are similar to the way 'Twitter Places' [104] currently adds geo-location context to tweets.) Sensor data annotations would add to the semantic annotation described by Sheth [64] and aid in analysis and dissemination of information.

### 3D Town Project - three-dimensionalisation of indoor and outdoor surveillance networks

In recent years, urban ICT (Information and Communication Technology), (such as mobile and wireless communication technologies and the Internet, ubiquitous sensing and computing, digital media and urban screens), has been rapidly modifying city life. The 'augmented city' is a modern definition of urban spaces, where no discernible boundaries exist between "virtual" and "physical" spaces [105]. The "virtual" urban space is a digital environment where the urban ICT is connected to "physical" urban space. The "virtual" urban space is often represented by three-dimensional (3-D) cityscape models. These 3-D city models are virtual representations of the "physical" urban spaces that digitally reproduce all the urban objects with a semantically enriched polyhedral world. Human beings' physical-digital intersection exists through the awareness of their location (sense of belonging to place) on the global level, but also on a very local scale. This location awareness does not only mean having knowledge of 'where we are', but also the ability to perceive or to be conscious of objects, events and patterns of surrounding environments with respect to 'where we are'.

Today's most representative augmented city examples are the GeoWeb portals such as Google Earth and Microsoft Bing Maps [106] that integrate location-based geographical information with an extremely broad range of non-spatial information through Web technology. Three-dimensional city models are a crucial extension of the GeoWeb since they provide an enriching, interactive, and visually appealing user experience that is "in tune" with users' natural visual-based thinking, imagination and space perception. Due to their many potential benefits in various domains, there has been a rapidly increasing demand for GeoWeb applications. Today's GeoWeb, however, is only visualising a static virtual world, which limits its use in applications where having knowledge of object dynamics/real-time motion is important. Our cities are dynamic, living environments with various kinds of real-time information and urban objects (people and vehicles) moving around us every day. To bring such dynamic information into the GeoWeb will give users a more immersive context to feel and interpret a real dynamic world.

A research team at York University, Canada, has been investigating and developing '3D Town', a research project that focuses on the development of an augmented city for sensing, distributing, interpreting and visualising the real-time dynamics of urban life. A core idea of the project is to integrate temporal information such as the movement of pedestrians and cars obtained using surveillance video with 3-D city models and thus implement a location-based awareness and means of interacting with 3-D city models through moving objects. To achieve this goal, the research team has implemented a dynamic GeoWeb ('D-GeoWeb') system that enables the management of [107]:

• Real-time data acquisition of wireless sensors distributed in indoor environment (*sensor layers*);

• Data protocols to integrate SensorML, IndoorML [108] and CityGML [109] (*protocol layers*);

• Data management layers to handle indoor and outdoor 3-D models and associate semantics, real-time sensor and moving object information (*data layers*); and

• Dynamic response to and rendering of requests by tracked moving objects (*responsive layers*).

**Figure 12:**
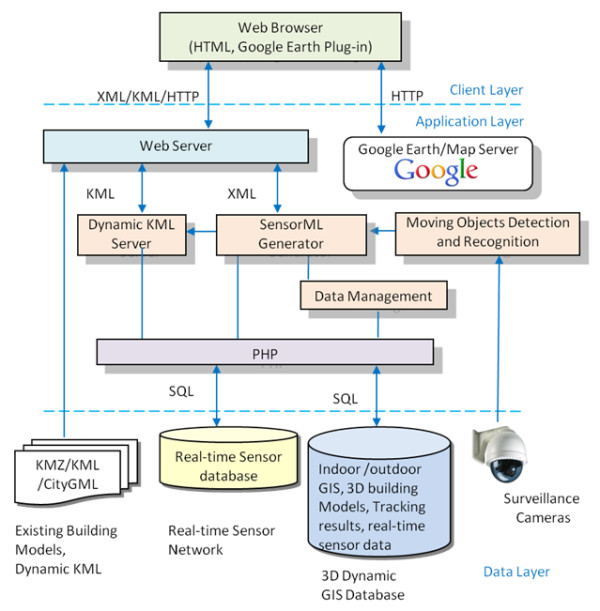
**Dynamic GeoWeb server**. KML: Keyhole Markup Language; KMZ: zipped KML; PHP: Hypertext Preprocessor (a server-side scripting language); SQL: Structured Query Language.

A schematic of data flow in D-GeoWeb is shown in Figure 12. To avoid labour-centric processing bottlenecks, a new method has been investigated to integrate indoor and outdoor models using geometric hashing algorithm [110]. The proposed method aims to integrate 2-D floor plans with terrestrial laser scanning data in an automatic manner. Thus, a fully integrated 3-D building model was reconstructed to create a seamless outdoor and indoor navigation database. New object detection and motion tracking algorithms were developed to detect, classify and track people and cars using pan/tilt video sequences [111]. '3D Town' is able to co-register indoor and outdoor wireframes with surveillance videos in a combination of vanishing point detection and line matching between wireframes and line segments detected from the image frames. In addition, a vision algorithm was implemented to track and recognise moving objects from surveillance videos. Finally, detected moving objects were localised and rendered in 3-D models as a sprite on a real-time basis. A prototype Web-based system was developed based on Google Earth Plug-in [112] to visualise the dynamic information and provide an interface between the users and the dynamic virtual 3-D world (but a WebGL [113] implementation would have been ideal, for plugin-free Web browser access of the dynamic 3-D virtual globe, e.g., [114,115]).

**Figure 13:**
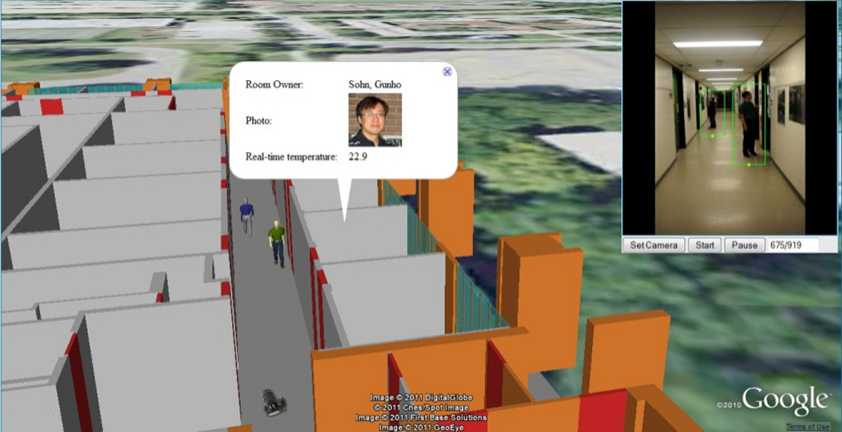
**3D Town indoor tracking and location intelligence: video from surveillance camera (upper-right inset)**. The results of tracking people are visualised using 3-D sprites. Wireless sensor data are dynamically read as the sprite accesses them.

**Figure 14:**
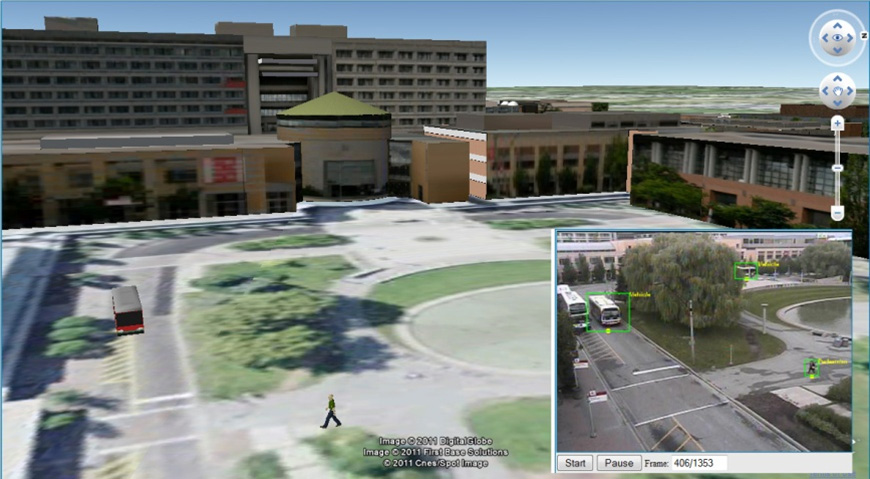
**3D Town outdoor moving object tracking and recognition: video from surveillance camera (bottom-right inset)**.

The 3D Town research shows promising results and demonstrates the feasibility of integrating dynamics and movements detected from sensors and videos with a corresponding 3-D virtual world. Figure 13 demonstrates 3D Town's capability of tracking people, who walk in a hallway, using surveillance video (upper-right inset) and localising them with 3-D sprites in Google Earth. A balloon shows a communication (room's occupancy and temperature information) between the virtual sprite and Crossbow wireless sensor [116], which pops up when the sprite stops at a room. Figure 14 presents results of tracking buses and pedestrians using surveillance camera under outdoor circumstances, which are again localised with 3-D sprites in Google Earth.

### PIEs - Precision Information Environments for emergency management

The first decade of the 21^st ^century saw a range of disasters strike worldwide, including terrorist attacks, hurricanes, tsunamis, wildfires, earthquakes, and a pandemic. The immediate and far reaching impacts of these disasters highlight the need for rapid and effective emergency management. The immense tragedy, uncertainty, and fear generated by an emergency underscore the necessity for effective regional preparation, response, recovery, and restoration.

For emergency management personnel to make accurate and timely decisions, they must have situational awareness, an accurate perception of the situation that they are facing and its complex data-scape. Essential to good situational awareness is the ability to provide relevant and timely information to decision makers and the public. Effective information collection and sharing has long been recognised as a challenge in emergency management [117].

These issues are growing more difficult with the adoption of social media as a pervasive way to share and disseminate information. The public is rapidly becoming the first reporter in the field to capture and disseminate information about an event as it occurs. Emergency management personnel increasingly need to leverage social media for situational awareness and information sharing. However, the volume, potential for anonymity, and lack of context can make information derived from social media hard to trust, and its intent and origin hard to discover.

As a point of reference for data volumes in social media (the 'problem space'), in just one hour Facebook [118] has 5,553,000+ status updates, 30,624,000 comments and 8,148,000 photos (statistics as of January 2011) [119]. In the same hour, Twitter has about 10,416,000 new tweets (statistics as of October 2011) [120], Flickr has 180,000 new photos (statistics as of September 2010) [121] and YouTube has another 2880-hour worth of video (statistics as of 2011) [122]. This is an information rich society and one where anyone can publish or broadcast. All of this information can be leveraged for situational awareness in an emergency, but no one user or group can digest it all.

The fast pace and critical nature of emergency management requires the ability to access and share information efficiently and effectively. Personnel often have difficulties obtaining the information they need for an effective response, and they frequently find that information is not shared across organisations. With the addition of social media as another input, there is often more information available than can be understood at the pace needed (information overload - *cf*. the notion of the Social Web "firehose"). To address this challenge, mechanisms must be put in place to assure that personnel always have awareness of, and trust in, the information relevant to their current role and activity.

Researchers at Pacific Northwest National Laboratory (PNNL) are developing future work environments (situation rooms) for the emergency management community that enable high-volume data feeds, including social media, to be processed and analysed such that participants in a response activity receive tailored data relevant just to their needs and roles. These Precision Information Environments (or PIEs) provide tailored access to information from disparate sources augmented with decision support capabilities in a system that is designed around the multiple user roles, contexts, and phases of emergency management, planning, and response. A Precision Information Environment provides visual analytic [123] capabilities through novel interactions that transform the way emergency professionals - from first responders to policy makers - engage with each other and with information. To develop the requirements for these environments, PNNL adopted a user-centred design philosophy in which emergency management personnel are involved in all aspects of the research from requirements definition, to scenario and use case development, constructing a capability vision, and feature development. The research team performed over fifty interviews, contextual inquiries and ethnographic studies to better understand the emergency management community and document the gaps and opportunities in the space. This work led to a scenario-driven vision articulated through a research agenda and video [29,30].

**Figure 15:**
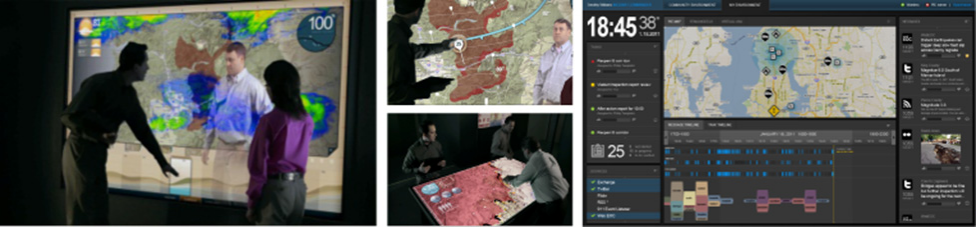
**Spatially-enabled elements of the Precision Information Environments (PIEs) vision**. From left: collaborative modelling and decision making, natural user interactions, and role- and task-driven information and map displays that synthesise large amounts of heterogeneous data about an event into a user-specific display.

This vision video (Figure 15), designed around a wildfire scenario 8-10 years into the future has become a north star for development, stakeholder outreach and user feedback. The vision for PIEs integrates data modelling and visual analysis, real-time and historical data streams, and novel user interface concepts in an approachable, easy to use collaborative application. Information and visual interfaces are tailored to the specific roles and tasks of each user. The goal is real-time synthesis, communication, and analysis of dynamically generated and collected information, all of which provides for sound decision making. PIEs allow users to engage with each other and leverage their collective expertise and experience in an environment combining virtual worlds based on actual physical models, real-time, multimodal data streams, and sophisticated visualisation tools to support actions, assessments, and decision-making (*cf*. vision in [27]).

#### Global profile, dynamic reputation and trust

Reputation and trust, both of emergency management personnel and members of the public that provide data, are equally important. Emergency management personnel often form *ad hoc *teams or networks during an event in which not all parties or individuals are known to each other. Emergency managers must also often rely on third party public reporting to assess the situation before responders are dispatched to the scene. In some cases, as in a natural disaster, an event can be so large and remote that professional assessment is not possible (in a timely manner). In those cases, profile and source assessment is just as important as the situation. Is the source trustworthy? Is there prior experience to suggest how useful a source's input is likely to be?

The global profile system inside a Precision Information Environment is applied to emergency management professionals and PIE content sources such as government professionals, nongovernmental organisations, private industry, and citizens. Each type of source is assigned a reputation score that is adapted over time based on the content they create (and how it is used by others) and the people they interact with. Emergency management professionals and responders may initially receive the highest reputation score followed by other government professionals that use or could use the environment. Other sources of content may initially have less trust associated with the data they provide, but their reputation score can increase based on other users' rating of their contributions.

The system also allows for source tracking and measuring the effectiveness of a message. It can also track a message as it changes or distorts through the social network, allowing emergency management to isolate when a message deteriorates. They can then reinforce the message through the social network by releasing an update to answer new questions and theories as they arise.

#### Tailored information services and adaptive data triage

At the centre of the Precision Information Environment is a profile for each user that defines the user's information interests and needs. One's role in an emergency event is a core part of this profile, and the PIE system uses roles defined by the National Incident Management System of the US Federal Emergency Management Agency to provide an initial template for information interest. Role-driven tailored information services and adaptive data triage bring 'Precision' into a Precision Information Environment. They allow an emergency manager to get exactly the right information at the right time and avoid information overload by filtering data to only those which are likely to be most relevant to a given user. In this way, the user can stay focused on the tasks and activities that matter.

**Figure 16:**
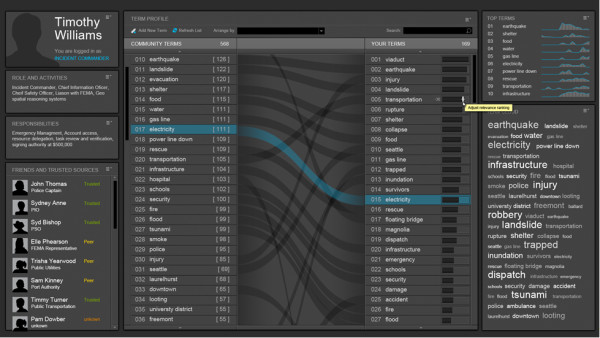
**PIEs user profile and signature ranking system showing term relevance**. At the centre of the display is a mapping between prioritised concepts in a user's profile (right) and concepts aggregated across all users' profiles (left).

PNNL's approach to information triage relies on a "signature" for each user that defines the terms that they are interested in and the relative weight of each term. As new data arrive from social media and other sources, a similar signature is constructed for each new data item. By comparing user and item signatures, we can determine which items are most relevant to which users. The initial recommendations made by the PIE system are tuneable by the user, so that as the situation changes so can his or her environment. Data are filtered and sorted based on the relevancy score for each user. This makes precision possible and allows the user to focus on content that is personally of interest to him/her based on his/her current activity. As the user works, communicates and shares information, he/she can train the system about his/her interests by simply 'voting' on whether or not each object is of interest to him/her (Figure 16). If an object is interesting, then those signatures get added to the user's profile (or weights increased if already in the profile). If it is not relevant, the ranking of the signatures gets diminished and gets a lower relevancy score.

PNNL uses a technique called 'tailored information services' to provide relevance-ranked data to every component of a PIE system. In addition to managing data ingest and triage, PIE tools can support creation and tracking of tasks in emergency response. All events, including data and tasks, are passed through the core PIE relevance engine, so that all information, regardless of source, can be accessed through a middle layer that performs per-user triage.

#### Collaborative natural user interfaces

In addition to data overload, there can be 'software (user interface) overload' in the emergency management community. Emergency management personnel are inundated with many different applications, each of them potentially specialised to activities that are only exercised during an emergency. As a result, they have to learn or retrain to use the software when an emergency occurs, which hinders adoption and use of new technology. The community is also highly mobile and is frequently marked by movement between user environments, such as from the desktop, to a collaborative setting, to the field. As a result, interfaces to data analysis and collaboration systems must be consistent across these platforms to minimise the learning curve.

A Natural User Interface (NUI) refers to a user interface that feels invisible, or becomes invisible with successive learned interactions, to its users. These interfaces are "natural" because the interactions they use are intuitive in their consistency with actions people already use outside of computer interfaces, e.g., touch, gesture and voice [124]. PIEs use NUI principles to allow users, especially those unfamiliar with a new system, to learn how to extract value from an information display quickly, and to create a desirable and engaging user experience.

**Figure 17:**
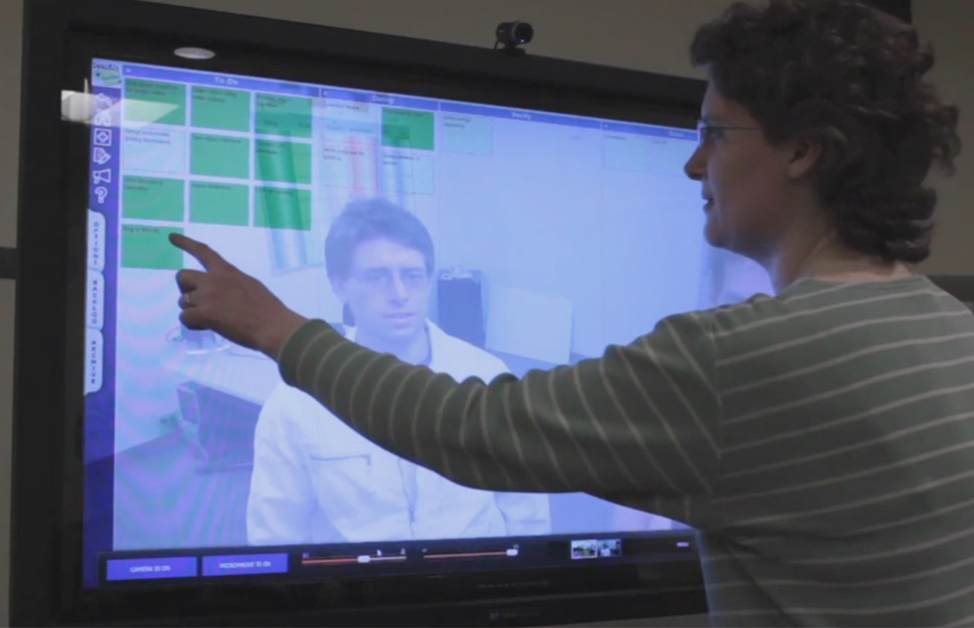
**LiveWall prototype in use during a usability study at PNNL**.

PNNL is also actively researching and prototyping new video teleconferencing devices that are designed to be 'always on' and support *ad hoc *meeting and information discovery among geographically distributed teams. LiveWall (Figure 17) is a prototype that supports high-definition, full-size video overlaid with an adaptable transparent information layer that gives the user the feeling of looking through a digital glass panel into another room. LiveWall uses a multitouch interface to allow distributed teams to interact with data and engage in collaborative decision making in a manner very similar to how the team would work if it were co-located and discussing a shared display (such as a printed map) in front of them.

## Discussion and conclusions

Increasing numbers of gadgets and appliances, including medical and hospital diagnostic devices, are now Internet-connected or embedding M2M SIM cards/GSM (Global System for Mobile Communications/Groupe Spécial Mobile) modules to allow reporting data to backend systems for diagnostic, telemetry and control purposes, and to gain useful insights about the populations using such devices. While it is possible to track and monitor the behaviour of crowds of people without even letting them know that they are being watched, e.g., tracking population movements using mobile phone network data (SIM card movements) during disasters and outbreaks [125,126], analysing aggregates of data generated during patient visits to clinics/hospitals, diagnostic laboratories and pharmacies [127], or using Internet search engine analytics [128-130] (though the latter method should always be interpreted with a grain of salt, as it might not work equally well for all conditions, the findings might not always be accurate [131], and search engine query words and phrases tend to lack a clear and complete context attached to them and to be less "expressive" than other forms of textual expression using social media), the real power and uniqueness of crowdsourcing lies in the active participation of intelligent humans in a task assigned to them. People, as social beings, have always shared information and helped one another in various situations; social media and crowdsourcing capitalise on this fact and enable us to share and support more (both in quantity and frequency, more content, more often), with many more people and much more quickly. Kamel Boulos' 2005 phrase-term 'wikification of GIS by the masses' [1] conveys this meaning by comparing the process to that of collaboratively editing a wiki such as Wikipedia [132] by a distributed network of a large number of intelligent human editors, where the power of darwikinism [76] comes into play. Volunteer citizens acting in groups (crowds) and sharing communication horizontally (in addition to vertically 'up' and 'down', as necessary) are challenging the notion that rapid information flow 'upwards' provides the optimal configuration. Information shared horizontally has been shown to be:

• *More timely*, as there is a peer pressure to help equals;

• *More complete*, as there is transparency in the reporting;

• *Higher in quality (in terms of sensitivity)*, as local populations know what is the baseline behaviour of their own communities; and also as peers sensitise each other to be on the look for certain 'signals' (for example, one volunteer reports high numbers of children absent from schools, prompting others in the network to ask the same question in their vicinity); and

• *Higher in quality (in terms of specificity)*, as being local allows to quickly validate, verify information, and dismiss false positives earlier (cross-validation and verification by peers).

The technologies supporting 'citizens as sensors' have to be:

• *Horizontal*, allowing citizens to share information with each other, in addition to vertically with situation rooms and similar centres (e.g., Amazon's Price Check comparison-shopping app [133] allows sharing in-store prices vertically with Amazon, as well as horizontally with other customers via Twitter, Facebook and text messages, in addition to accessing other customers' product reviews);

• *Semi-structured*, allowing people to report what they deem relevant, not restricting them to very tightly structured forms and templates;

• *Real-time*, as information has to flow back and forth in real-time allowing the creation of dialogue;

• *Open*, whereby members of a group have to feel they can add to the conversation or reporting task other people who can provide value, thus creating a meritocracy and allowing the crowdsourced community to grow while factoring 'credentials, reputation and trust' into the process;

• *Geo-aware*, noting that local citizens have a high authority on what is nearby to them; having this piece of information is essential for the contextualisation and interpretation of data; and

• *Accessible*, meaning that the process can also work on even the simplest mobile phone, for example using SMS text messages (*cf*. GeoSMS).

The presence of multimodal sensors on more advanced smartphones and tablets carried by citizens is also enabling a broad range of possibilities, but the automatic collection of detailed sensor data from mobile devices may compromise user privacy and this has to be adequately addressed in mobile participatory sensing applications relying on such data [134]. In an editorial for a 2008 special issue of *GeoJournal *on citizen-contributed geographic information, Elwood raises some of the more sociological issues regarding crowd-enabled technologies that are equally worth considering [135].

The counterpart of crowdsourcing is 'crowdreaching', which involves reaching out to people with various messages, e.g., 'health tips', especially at times of mass stress [75], and capitalising on the viral and ubiquitous natures of mobile and social media to do so (cyberinfluence). Consumers and citizens might also be willing to pay for useful and individualised information. Services that provide health tips can thus be commercially viable (if not funded by government/public health authorities or similar bodies), in addition to being a direct source of self-reported health information. For example, using tools such as InSTEDD RemindEm [136] (free and open source), pregnant citizens can subscribe to health tips for their current stage of pregnancy by texting in their LMP (Last Menstrual Period); location-tailored tips for diabetics, pre-operatory or post-operatory guidelines, etc. can also be served using RemindEm. Platforms that offer some crowdreaching value and crowdsharing opportunities to citizens reporting their health conditions (while adequately protecting users' privacy) such as PatientsLikeMe [137] allow the creation of crowdsourced aggregated datasets that shed light on specific populations, diseases and geographies with regards to health.

For cross-border health surveillance, where neighbouring towns/cities and countries affect each other's health but might be very 'distant' in terms of the bureaucratic processes required to share information in a timely manner (even when all essential data sharing agreements are in place [138]), tools are needed that respect each jurisdiction's need for controlling access to its crowdsourced (expert) reports and data, while providing mechanisms for sharing adequate situational awareness of health events in sentinel border sites. Some of this functionality is available in Veegilo [139], a simple open source tool that aggregates disease indicator numbers from national databases into a common space where incidence and reported deaths of monitored diseases can be seen and compared across sentinel sites and over time. Its primary use has been for international health networks, but can also be applied at national or provincial level to simplify data entry.

In this paper, we presented a comprehensive state-of-the-art review of the overlapping domains of the Sensor Web, citizen sensing and 'human-in-the-loop sensing' in the era of the Mobile and Social Web, and the roles these domains can play in environmental and public health surveillance and crisis/disaster informatics. We covered the key issues and trends in these areas, the challenges faced when reasoning and making decisions with real-time crowdsourced data (such as issues of information overload, "noise", misinformation, bias and trust), the core technologies and Open Geospatial Consortium (OGC) standards involved (Sensor Web Enablement and Open GeoSMS), as well as a few outstanding project implementation examples from around the world (Common Scents/the Copenhagen Wheel, Linked Sensor Middleware, 3D Town, and Precision Information Environments).

## Competing interests

The authors declare that they have no competing interests.

## Authors' contributions

MNKB conceived and drafted the manuscript with equal (in terms of importance and/or word count) contributions and expert insight from BR, DNC, JGB, GS, RB, WAP, EJ and K-YSC. MNKB also conducted the core literature review, identified and reflected on the main trends in the field, located and invited the co-authors on this paper, and edited their contributions. All authors read and approved the final manuscript. Commercial products and company/brand names mentioned in this paper are trademarks and/or registered trademarks of their respective owners.
